# Trends in Positive *BRCA* Test Results Among Older Women in the United States, 2008-2018

**DOI:** 10.1001/jamanetworkopen.2020.24358

**Published:** 2020-11-05

**Authors:** Fangjian Guo, Matthew Scholl, Erika L. Fuchs, Rebeca Wong, Yong-Fang Kuo, Abbey B. Berenson

**Affiliations:** 1 Department of Obstetrics and Gynecology, The University of Texas Medical Branch at Galveston, Galveston; 2 Center for Interdisciplinary Research in Women’s Health, The University of Texas Medical Branch at Galveston, Galveston; 3 School of Medicine, The University of Texas Medical Branch at Galveston, Galveston; 4 Department of Preventive Medicine and Community Health, The University of Texas Medical Branch at Galveston, Galveston; 5 Office of Biostatistics, Department of Preventive Medicine and Community Health, The University of Texas Medical Branch at Galveston, Galveston; 6 Institute for Translational Science, The University of Texas Medical Branch at Galveston, Galveston

## Abstract

**Importance:**

Genetic testing for *BRCA1/2* pathogenic variants has been used for targeted, individualized cancer prevention and treatment. A positive *BRCA* test result indicates a higher risk for developing *BRCA*-related cancers. During the past decade, testing criteria have loosened. The impact of these loosened criteria on *BRCA* testing in older women has not previously been studied.

**Objective:**

To assess whether the rate of positive *BRCA* test results changed between 2008 and 2018 among older women in the United States.

**Design, Setting, and Participants:**

This cross-sectional study used a 10% random sample of women 65 years of age or older from Optum’s deidentified Integrated Claims-Clinical data set (2008-2018), a large national electronic health record data set. A total of 5533 women with *BRCA* test results from January 1, 2008, to March 31, 2018, were evaluated.

**Main Outcomes and Measures:**

Annual percentage change in positive *BRCA* test results was evaluated. Multivariable logistic regression models were used to assess the association between positive test results and race/ethnicity, region of residence, income, educational level, and personal history of breast or ovarian cancer.

**Results:**

Of 5533 women 65 years of age or older (mean age, 68.1 years [95% CI, 67.9-68.4 years]) who underwent *BRCA* testing from 2008 to 2018, most (4679 [84.6%]) were non-Hispanic White women, and 1915 (34.6%) resided in the Midwest. Positive *BRCA* test results decreased from 85.7% (36 of 42) in 2008 to 55.6% (140 of 252) in 2018 (annual percentage change, −2.55; 95% CI, −3.45 to −1.64). Among patients with breast or ovarian cancer, positive test results decreased from 83.3% (20 of 24) in 2008 to 61.6% (61 of 99) in 2018, while among women without breast or ovarian cancer, positive test results decreased from 87.5% (21 of 24) in 2008 to 48.4% (74 of 153) in 2018 (annual percentage change, −3.17 vs −2.49; *P* = .29). Women with positive test results were more likely to be non-Hispanic Black women, to live in the West or South, to live in areas with a low percentage of college graduates, or to not have a personal history of breast or ovarian cancer.

**Conclusions and Relevance:**

This study suggests that there was a significantly decreasing rate of positive *BRCA* test results among women 65 years of age or older. Socioeconomic and regional disparities in testing use remain an issue.

## Key Points

**Question:**

What are the trends in positive *BRCA* test results among older women in the United States from 2008 to 2018?

**Findings:**

This cross-sectional study of 5533 women aged 65 years or older from a large national electronic health record data set found that the rate of documented positive *BRCA* test results significantly decreased from 2008 to 2018, especially among patients with breast or ovarian cancer. Women with positive test results were more likely to be non-Hispanic Black women, to live in the West or South, to live in areas with a low percentage of college graduates, or to not have a personal history of breast or ovarian cancer.

**Meaning:**

The rate of positive *BRCA* test results has continuously decreased, which may be partly explained by loosening of testing criteria over time.

## Teaser

This cross-sectional study assesses whether the rate of positive *BRCA* test results changed between 2008 and 2018 among older women in the United States.

## Introduction

The discovery of *BRCA*1 (OMIM 113705) and *BRCA*2 (OMIM 600185) pathogenic variants in many patients with breast and ovarian cancer^1^ led to the recommendation of genetic testing for targeted and individualized cancer prevention and treatment.^1,2,3,4^ Professional organizations, including the US Preventive Services Task Force and the National Comprehensive Cancer Network, have issued *BRCA* testing recommendations for women whose racial/ethnic backgrounds, personal histories, or family histories are associated with an increased risk for carrying *BRCA* pathogenic variants.^3,4^ A previous study reported on trends in the use of *BRCA* testing in patients with cancer and unaffected women among US adult women aged 20 to 65 years using claims data from the Clinformatics Data Mart Database.^5^ A gradual shift was observed as *BRCA* testing moved from being used mainly in women who already had a diagnosis of breast or ovarian cancer to being used for cancer prevention. The current study evaluates how the rate of documented positive results for *BRCA* testing changed over time and also evaluates the association between clinical and demographic characteristics and positive test results among US women 65 years of age or older.

## Methods

This cross-sectional study used data from a 10% random sample of women aged 65 years or older in Optum’s deidentified Integrated Claims-Clinical data set (2008-2018). As of 2018, this data set integrated 85 health systems and represented more than 140 000 practioners for a cumulative 91 million lives across all 50 states. These longitudinal electronic health record (EHR) data contain deidentified information on demographic characteristics, diagnosis, hospitalizations, laboratory test results, medications, observations, outpatient visits, clinician notes, and procedures. In addition, natural language processing software can be used to extract important clinical information from clinician notes. The data set is broadly geographically and demographically representative with similar compositions in race/ethnicity, sex, age, and income to the US population with private medical insurance. This study was exempted from full board review by the institutional review board at The University of Texas Medical Branch as the data were deidentified. This report follows the Strengthening the Reporting of Observational Studies in Epidemiology (STROBE) reporting guideline for observational studies.

To be included in the sample used for analysis in this study, participants must have been women 65 years of age or older with *BRCA* testing results from January 1, 2008, to March 31, 2018, from the 10% random sample in Optum’s deidentified Integrated Claims-Clinical data set. We included all 5533 women 65 years of age or older with *BRCA* testing results from 2008 to 2018. We did not exclude any of those women. The *BRCA* test results were obtained from laboratory test results in the EHR and were also extracted from clinician notes using natural language processing. Positive test results indicated the presence of pathogenic variants. The association between positive test results (a binary variable) and race/ethnicity, region of residence, educational level, income, and personal history of breast or ovarian cancer was evaluated. Race/ethnicity was self-reported and classed as non-Hispanic White, non-Hispanic Black, Hispanic, and other. Region of residence was divided into South, West, Midwest, and Northeast according to US Census regions. Zip code data were used to assess the percentage of people with at least 1 college degree and annual household income. We treated income and educational level as binary variables and used commonly adopted cutpoints of $50 000 for annual household income and 25% college educated for educational level. Cases of previously diagnosed breast cancer were identified by the *International Classification of Diseases, Ninth Revision* (*ICD-9*) code V10.3 and the *International Statistical Classification of Diseases and Related Health Problems, Tenth Revision* (*ICD-10*) code Z85.3. Personal history of malignant neoplasm of the breast was identified by *ICD-9* code 174.x and malignant neoplasm of the breast by *ICD-10* code C50. Carcinoma in situ of the breast was identified by *ICD-9* code 233.0 and *ICD-10* code D05. Previously diagnosed ovarian cancer was identified by *ICD-9* code 183.0 and *ICD-10* code C56 for malignant neoplasm of ovary and *ICD-9* code V10.43 and *ICD-10* code Z85.43 for a personal history of malignant neoplasm of ovary. Family history of ovarian cancer was identified by *ICD-9* code V16.41 and *ICD-10* code Z80.41. Family history of breast cancer was identified by *ICD-9* code V16.3 and *ICD-10* code Z80.3.

### Statistical Analysis

Documented positive test result rates were calculated by using women with positive test results noted in their EHR during each year as the numerator and the female population with documented *BRCA* test results that year as the denominator. Next, documented positive test result rates were assessed for linear trends. Then, the annual percentage change (APC) of total *BRCA* testing and the APC of test results were calculated. The APC was calculated as [exp (β) − 1] × 100, where the regression coefficient (β) was estimated by fitting a least-squares regression line to the natural logarithm of the rates, using the calendar year as a regressor variable. We assessed changes in annual rates of positive *BRCA* test results by region of residence and personal history of breast or ovarian cancer. Women with and women without breast or ovarian cancer may have different rates of positive *BRCA* test results, as the purpose of *BRCA* tests in these 2 groups of women are different,^5,6^ for cancer treatment and prevention, respectively. Differences in mean age and percentage of college education were assessed by the Wald *t* test. Other differences in characteristics between women with and women without positive test results were assessed by the χ^2^ test. The association between positive test results and race/ethnicity, region of residence, educational level, income, and personal history of breast or ovarian cancer was assessed by fitting a model with all those variables. To account for the fact that educational level and annual household income were aggregated databased on the zip code data, we fitted a hierarchical logistic regression model and treated the zip code effects as random effects only. We also assessed whether mastectomy types differed between patients with breast cancer with or without positive *BRCA* test results to explore whether *BRCA* test results changed the treatment plan. We performed additional sensitivity analyses by assigning sample weights to individuals in each of 4 US regions such that the study population was composed of representative proportions of women from the 4 US regions as the 2010 US standard population (Northeast, 19.4%; Midwest, 22.4%; South, 37.0%; and West 21.2%). Missing data in region of residency were categorized as unknown (n = 94). Statistical analyses were conducted using SAS software, version 9.4 (SAS Institute Inc), and a 2-sided *P* < .05 was considered statistically significant for the purposes of our analysis.

## Results

A total of 5533 women aged 65 years or older (mean age, 68.1 years [95% CI, 67.9-68.4 years]) had *BRCA* testing results noted in their EHRs between 2008 and 2018 (Table 1). Most women (4679 [84.6%]) were non-Hispanic White and 1915 (34.6%) resided in the Midwest. Among the 1502 women without positive test results, 45 (3.0%) were non-Hispanic Black women and 708 (47.1%) resided in the Midwest. Among the 4031 women with positive test results, 228 (5.7%) were non-Hispanic Black women, and 1207 (29.9%) resided in the Midwest.

**Table 1.** Characteristics of Adult Women Who Had *BRCA* Test Results Noted in their Electronic Health Records in 2008-2018

Characteristic	Women, No. (%) [95% CI]			*P* value^a^
	Total (N = 5533)	No positive test result (n = 1502)	Positive test result (n = 4031)	
Age, mean, y	68.1 (67.9-68.4)	68.5 (68.1-68.9)	68.0 (67.7-68.3)	.05
Race/ethnicity^b^				
Non-Hispanic White	4679 (84.6) [83.6-85.5]	1340 (89.2) [87.6-90.8]	3339 (82.8) [81.7-84.0]	<.001
Non-Hispanic Black	273 (4.9) [4.4-5.5]	45 (3.0) [2.1-3.9]	228 (5.7) [4.9-6.4]	
Hispanic	96 (1.7) [1.4-2.1]	19 (1.3) [0.7-1.8]	77 (1.9) [1.5-2.3]	
Region^c^				
Northeast	1132 (20.5) [19.4-21.5]	407 (27.1) [24.8-29.3]	725 (18.0) [16.8-19.2]	<.001
West	1403 (25.4) [24.2-26.5]	161 (10.7) [9.2-12.3]	1242 (30.8) [29.4-32.2]	
Midwest	1915 (34.6) [33.4-35.9]	708 (47.1) [44.6-49.7]	1207 (29.9) [28.5-31.4]	
South	989 (17.9) [16.9-18.9]	198 (13.2) [11.5-14.9]	791 (19.6) [18.4-20.8]	
Percentage with college education^c^	5439 (27.2) [26.9-27.4]	422 (28.7) [28.2-29.1]	1055 (26.6) [26.3-26.9]	<.001
Annual household income ≥$50 000^c^	5439 (24.2) [23.0-25.3]	479 (32.5) [30.1-34.9]	836 (21.1) [19.8-22.4]	<.001
Family history of breast cancer	5533 (34.1) [32.8-35.3]	573 (38.1) [35.7-40.6]	1312 (32.5) [31.1-34.0]	<.001
Family history of ovarian cancer	5533 (8.2) [7.5-8.9]	117 (7.8) [6.4-9.1]	337 (8.4) [7.5-9.2]	.49
Personal history of breast cancer	5533 (39.0) [37.8-40.3]	685 (45.6) [43.1-48.1]	1475 (36.6) [35.1-38.1]	<.001
Personal history of ovarian cancer	5533 (6.6) [5.9-7.2]	93 (6.2) [5.0-7.4]	270 (6.7) [5.9-7.5]	.50

^a^ The differences in characteristics between women with and women without pathogenic variants were assessed by the χ^2^ test, except for differences in mean age and percentage of college education, which were assessed by the Wald *t* test.

^b^ Race/ethnicity in the “Other” category was not reported (n = 322).

^c^ Region of residence in the “Unknown” category was not reported (n = 94). These participants also had missing values for “Percentage with college education” and “Annual household income ≥$50 000,” as the latter 2 variables were derived from zip code data.

**Figure 1.**
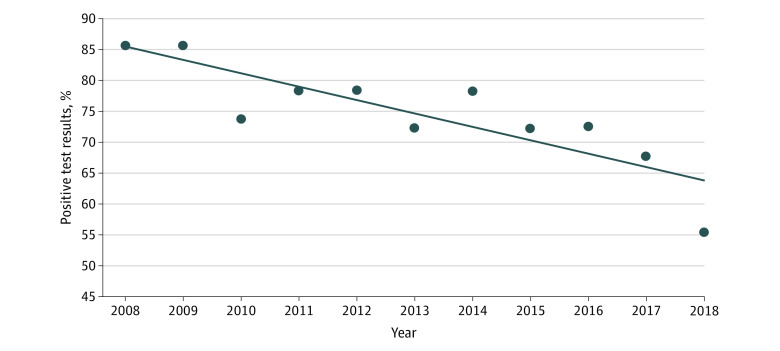
Documented Positive *BRCA* Test Results Among Women 65 Years of Age or Older, 2008-2018 The diagonal line indicates the trend in documented positive *BRCA* test results.

**Figure 2.**
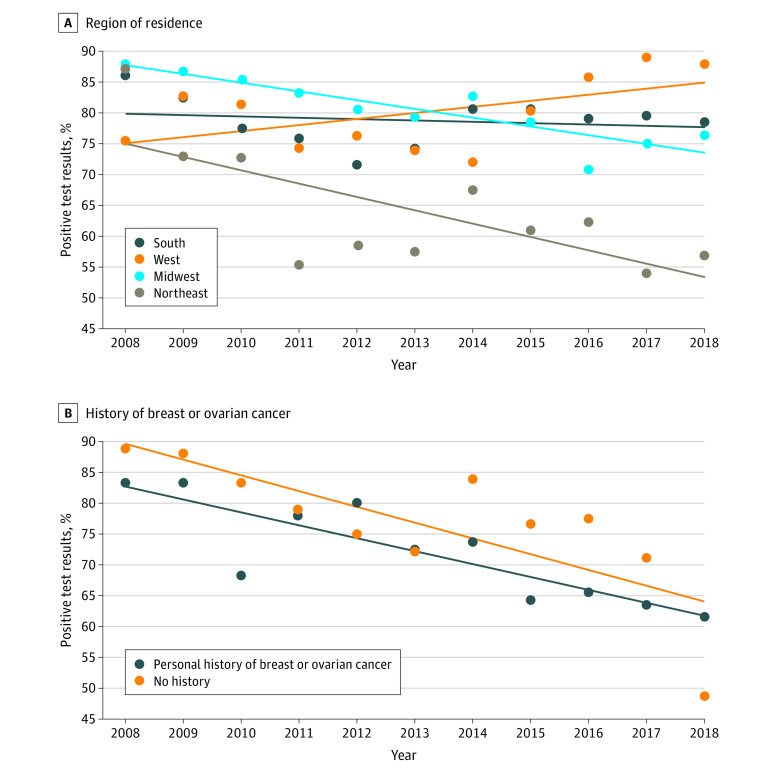
Documented Positive *BRCA* Test Results by Region of Residence and Personal History of Breast or Ovarian Cancer, 2008-2018 A, Region of residence. B, Personal history of breast or ovarian cancer. The diagonal lines indicate the trend in documented positive *BRCA* test results.

Documented positive test results decreased from 85.7% (36 of 42) in 2008 to 55.6% (140 of 252) in 2018 (APC, −2.55; 95% CI, −3.45 to −1.64) (Figure 1). Rates of positive test results decreased among patients residing in the Midwest and the Northeast but increased among patients in the West (Figure 2A). Between 2008 and 2018, positive results decreased from 83.3% (15 of 18) to 61.6% (61 of 99) among patients with breast or ovarian cancer, compared with a decrease of 87.5% (21 of 24) to 48.4% (74 of 153) among patients without breast or ovarian cancer (APC, −3.17 vs −2.49; *P* = .29) (Figure 2B).

Women with positive test results were more likely to be non-Hispanic Black women (adjusted odds ratio [AOR], 1.52 [95% CI, 1.04-2.22]), live in the West (AOR, 2.67 [95% CI, 1.63-4.39]) or South (AOR, 1.80 [95% CI, 1.14-2.86]), live in areas with a low percentage of college graduates (AOR, 1.54 [95% CI, 1.10-2.17]), or not have a personal history of breast or ovarian cancer (AOR, 1.30 [95% CI, 1.12-1.50]) (Table 2). A sensitivity analysis using sample weights to produce representative proportions of women from the 4 US regions showed similar results (eTable in the Supplement). Among 343 patients with breast cancer who had documented medical claims within 1 year before or after their documented *BRCA* test results, there were no differences in mastectomy types between patients with or without positive test results (Table 3).

**Table 2.** Adjusted Odds Ratios for Having a Positive *BRCA* Test Result in 2008-2018 Among Women 65 Years of Age or Older

Characteristic	Women No.	Positive rate, No. (%) [95% CI]	Adjusted odds ratio (95% CI)^a^
Race/ethnicity^b^			
Non-Hispanic White	4595	3280 (71.4) [70.1-72.7]	1 [Reference]
Non-Hispanic Black	268	224 (83.6) [79.1-88.0]	1.52 (1.04-2.22)
Hispanic	94	75 (79.8) [71.7-87.9]	1.34 (0.77-2.35)
Region			
Northeast	1132	725 (64.0) [61.2-66.8]	1 [Reference]
West	1403	1242 (88.5) [86.9-90.2]	2.67 (1.63-4.39)
Midwest	1915	1207 (63.0) [60.9-65.2]	1.43 (0.94-2.18)
South	989	791 (80.0) [77.5-82.5]	1.80 (1.14-2.86)
Percentage of college degree^c^			
>25	3108	2122 (68.3) [66.6-69.9]	1 [Reference]
≤25	2331	1843 (79.1) [77.4-80.7]	1.54 (1.10-2.17)
Annual household income^c^			
≥$50 000	1315	836 (63.6) [61.0-66.2]	1 [Reference]
<$50 000	4124	3129 (75.9) [74.6-77.2]	0.96 (0.62-1.48)
Personal history of breast or ovarian cancer			
Yes	2383	1643 (68.9) [67.1-70.8]	1 [Reference]
No	3056	2322 (76.0) [74.5-77.5]	1.30 (1.12-1.50)

^a^ Adjusted odds ratios: the association between positive test results and race/ethnicity, region of residence, educational level, income, and personal history of breast or ovarian cancer was assessed by fitting a model with all those variables. Adjusted odds ratios were estimated by adjusting for other variables in the model.

^b^ Race/ethnicity in the “Other” category was not reported.

^c^ To account for the fact that educational level and annual household income were aggregated databased on zip code data, we fitted a hierarchical logistic regression model and treated the zip code effects as random effects only. Individuals in the “Unknown” category of region of residence were not included in the model (n = 94).

**Table 3.** Type of Mastectomy in Patients With Breast Cancer by *BRCA* Test Results (N = 343)^a^

Mastectomy type	No. (%) [95% CI]		*P* value^b^
	Positive test result (n = 235)	No positive test result (n = 108)	
Radical	40 (17.0) [12.2-21.9]	15 (13.9) [7.3-20.4]	.76
Simple	43 (18.3) [13.3-23.3]	21 (19.4) [11.9-26.9]	
Partial	152 (64.7) [58.5-70.8]	72 (66.7) [57.7-75.6]	

^a^ We included a total of 343 patients with breast cancer with *BRCA* test results who had medical claims within 12 months before or after documented *BRCA* test results in this analysis (after removing 3368 patients without breast cancer and 1822 patients without medical claims within 12 months before or after documented *BRCA* test results).

^b^ Based on χ^2^ test.

## Discussion

We used data from a large EHR data set to assess trends in documented *BRCA* testing results among older women. We observed a significantly decreasing documented rate of positive test results, which may be partly explained by a relaxing of testing criteria for testing women during the evaluated period. Evaluation of clinical guidelines issued by the US Preventive Services Task Force and the National Comprehensive Cancer Network demonstrates serially and consistently relaxed selection criteria for *BRCA* testing and genetic counseling over the years.^3,7,8,9,10^ These clinical criteria and practice guidelines for *BRCA* testing are focused mainly on patients with breast or ovarian cancer and rely on those patients as index patients to detect pathogenic variants in their relatives.^11,12,13,14^ In our study, the observed steady annual decrease in the rate of positive *BRCA* test results among patients with breast or ovarian cancer is potentially owing to the loosened criteria. *BRCA* testing in patients with early-onset breast or ovarian cancer can identify those with high-risk mutations, in whom specific treatment options may be needed.^15,16,17,18^ Patients with cancer who have positive test results for pathogenic *BRCA* variants could benefit from increased surveillance and risk-reducing measures to prevent other cancers for which their pathogenic variant places them at an increased risk. We recognize that this benefit is relatively small in our study population of older women in comparison with younger women. We also did not find any difference in mastectomy types between patients with breast cancer with or without positive *BRCA* test results.

Despite the decreasing rates of positive *BRCA* test results, socioeconomic and regional disparities existed in use of testing. We observed that, compared with non-Hispanic White women, non-Hispanic Black women were more likely to have a positive *BRCA* test result. The results of Hispanic women were not significant. Black and Hispanic women are usually found to underuse *BRCA* testing.^19^ Women included in our study were eligible for Medicare owing to their age, which may have increased accessibility of *BRCA* testing across racial/ethnic lines in ways not seen in younger women. Comparison with women younger than 65 years warrants further study. We recognize that this finding could also be owing to the relatively large and disproportionate number of non-Hispanic White women and relatively small sample size of minorities in the study and could be different if the numbers of Hispanic women were increased.

### Limitations

This study had several limitations. One main limitation is the generalizability of the data. Our data included more women in the Midwest compared with the standard US population, so while it was comprehensive across all 50 states, it was not proportionally representative. Our data were sourced exclusively from patients who went to health systems integrated into Optum’s EHR data and thus may not be applicable to women who visit other health systems. Further limitations include the potential for incorrect or missed documentation in the EHR. We studied only *BRCA* test results that were documented in the EHR; therefore, any undocumented *BRCA* test results, especially undocumented negative results, would be excluded from our calculations.

## Conclusions

The significantly decreasing rate of positive *BRCA* test results is evident among women 65 years of age or older, which may be partly explained by loosening of testing criteria during the evaluated time range. However, socioeconomic and regional disparities were identified and persisted in testing use.

## Notes

Supplement. **eTable.** Adjusted Odds Ratios for Having a Positive *BRCA* Test Result Using Sample Weights (n=5439)
