# Supplementary material for: Trends in Positive BRCA Test Results Among Older Women in the United States, 2008-2018
Source: JAMA Netw Open. 2020 Nov 5;3(11):e2024358. doi: 10.1001/jamanetworkopen.2020.24358 (PMC7645697; doi:10.1001/jamanetworkopen.2020.24358)
Supplement: Supplement. — eTable. Adjusted Odds Ratios for Having a Positive BRCA Test Result Using Sample Weights (n=5439) [file jamanetwopen-e2024358-s001.pdf]

Supplementary Online Content

Guo F, Scholl M, Fuchs EL, Wong R, Kuo YF, Berenson AB. Trends in positive *BRCA* test results among older women in the United States, 2008-2018. *JAMA Netw Open.* 2020;3(11):e2024358 doi:10.1001/jamanetworkopen.2020.24358

eTable. Adjusted Odds Ratios for Having a Positive *BRCA* Test Result Using Sample Weights (n=5439)

This supplementary material has been provided by the authors to give readers additional information about their work.

© 2020 Guo F et al. *JAMA Network Open.*

eTable. Adjusted Odds Ratios for Having a Positive *BRCA* Test Result Using Sample Weights (n=5439)

	n	Positive rate % (95% CI)	Adjusted odds ratios (95% CI)
Race/ethnicity[a]			
Non-Hispanic White	4595	73.6 (72.2-74.9)	Reference
Non-Hispanic Black	268	85.4 (80.8-90.0)	1.56(1.10-2.21)
Hispanic	94	80.4 (71.6-89.2)	1.28(0.75-2.19)
Region			
Northeast	1132	64.0 (61.2-66.8)	Reference
West	1403	88.5 (86.9-90.2)	2.70 (1.60-4.53)
Midwest	1915	63.0 (60.9-65.2)	1.39(0.89-2.18)
South	989	80.0 (77.5-82.5)	1.89(1.21-2.96)
Percentage of college degree			
>25	3108	69.5 (67.8-71.3)	Reference
≤25	2331	80.7 (78.9-82.5)	1.58(1.12-2.24)
Annual household income			
≥$50,000	1315	65.4 (62.7-68.0)	Reference
<$50,000	4124	77.5 (76.1-78.9)	0.93(0.59-1.44)
personal history of breast or ovarian cancer			
Yes	2383	70.0 (67.9-72.0)	Reference
No	3056	79.0 (77.5-80.5)	1.34(1.15-1.55)

We assigned sample weights to individuals in each four US regions such that the study population composed of representative proportions of women from the four US regions as the 2010 US standard population (Northeast 19.4%, Midwest 22.4%, South 37.0%, West 21.2%).

To account for that education and annual household income were aggregated data based on the zip code data, we fitted a hierarchical logistic regression model and treated the zip code effects as random effects only. Individuals in the ‘Unknown’ category of region of residence was not included in the model (n=94).

Adjusted odds ratios: The relationship between positive test results and race/ethnicity, region of residence, education, income, and personal history of breast or ovarian cancer was assessed by fitting a model with all those variables. Adjusted odds ratios were estimated by adjusting for other variables in the model.

[a] Race/ethnicity in the ‘Other’ category was not reported.

© 2020 Guo F et al. *JAMA Network Open.*
